# Working with public contributors to improve the patient experience at the Manchester Clinical Research Facility: an evaluation of the Experience Based Design approach

**DOI:** 10.1186/s40900-017-0059-x

**Published:** 2017-04-26

**Authors:** Kerin Bayliss, Rebecca Prince, Hal Dewhurst, Suzanne Parsons, Leah Holmes, Paul Brown

**Affiliations:** 1 Public Programmes Team, Research and Innovation Division, Central Manchester University Hospitals NHS Foundation Trust and the University of Manchester, Manchester Academic Health Science Centre, Manchester, UK; 2 Involvement and Engagement Steering Group, NIHR/Wellcome Trust Manchester Clinical Research Facility, Central Manchester University Hospitals NHS Foundation Trust, Manchester, UK; 3 NIHR/Wellcome Trust, Manchester Clinical Research Facility, Central Manchester University Hospitals NHS Foundation Trust, Manchester, UK

**Keywords:** Patient and public involvement, Co-design, Experience Based Design, Evaluation, Co-production

## Abstract

**Plain English summary:**

The Experience Based Design (EBD) approach involves patients, staff and members of the public working together to improve a service. This paper evaluates the methods that are used to involve patients and members of the public in a project that aimed to improve the patient experience at Manchester Clinical Research Facility (MCRF). The aim was to explore what helps staff and members of the public to work well together.

An evaluation questionnaire was used to get feedback from staff and public contributors. Questions included whether each person felt that they were able to shape the project; if they received enough training; whether they had enough time to complete each task; how well they thought the group worked together; and what could be improved. The findings showed that both staff and public contributors felt valued and that they were able to shape the project from the beginning. Training in EBD and research methodology, and providing enough time to complete each task helped to build relationships and increase confidence when contributing to the project. Personal benefits included a feeling of ownership over a worthwhile and rewarding project, increased awareness of public involvement and gaining new skills. The recommendations for successful involvement of patients and the public in EBD projects will hopefully be helpful for similar projects in the future.

**Abstract:**

**Background**

The Experience Based Design (EBD) approach promotes the effective involvement of patients and public contributors by enabling patients, public contributors and staff to co-design projects that aim to improve the patient experience. This approach allows patients and members of the public to have a role in shaping and improving current services. This paper aims to evaluate the EBD process from a public involvement perspective, exploring the barriers and facilitators to building successful working relationships.

**Methods**

An open-ended evaluation questionnaire was developed to gain feedback from staff and public contributors who co-produced an EBD project that aimed to improve the patient experience at Manchester CRF. Questions explored what worked well, how the project could be improved, and the benefits of being involved.

**Results**

Our findings highlight the importance of providing opportunities for staff, patients and members of the public to build relationships in order to feel confident in voicing their opinions. This can be achieved by training both staff and public contributors in EBD methodology to reduce any power imbalance that may exist. Negotiating adequate time to complete tasks and debate the best way forward also allows everyone to fully contribute to the project. Each individual felt that their contribution was valued and that they shaped the final action plan. Both public contributors and staff listed a number of personal benefits from their involvement in the project. This included a feeling of ownership over a worthwhile and rewarding project, increased awareness of public involvement in EBD projects and gaining new skills.

**Conclusion**

This evaluation provides recommendations for best practice for effectively involving public contributors in an EBD methodology. These findings aim to encourage a more consistent approach to EBD across organisations.

**Electronic supplementary material:**

The online version of this article (doi:10.1186/s40900-017-0059-x) contains supplementary material, which is available to authorized users.

## Background

The experiences that patients and the public have when they receive healthcare services are a valuable source of information that can be used to improve those services [1]. Information on these experiences is traditionally gathered using audits or surveys, which rely on staff setting specific questions which may miss what really matters to patients. For example, a score of 1-5 on a Likert scale may not fully reflect the complex nature of experience, emotions and expectations [1]. An Experience Based Design (EBD) approach goes beyond survey methods, promoting patient and public involvement in the process by enabling patients and staff to work in an active partnership to co-design projects that identify problems that can be practically overcome [1, 2]. This approach allows patients and members of the public to have a role in shaping and improving current services rather than simply being the final recipient [1, 2]. EBD traditionally involves patients but in this project public contributors were involved in designing an EBD project which explored how to improve patient experience in a clinical research facility.

EBD projects can involve gathering experiences from patients through in-depth interviewing or ‘storytelling’, observations and group discussions. Positive and negative ‘emotionally significant’ points are identified by patients, therefore focusing on experience and emotions rather than attitudes or opinions. Staff and patients then work together to prioritise areas for improvement, develop solutions and define key action points [2]. The two-way process can boost confidence and motivation levels, and the results often reveal unexpected areas for improvement that can be surprisingly simple to overcome. For example, it might identify that the most frustrating aspect of long waiting times relates to poor communication rather than the wait itself. This can lead to long-lasting change that genuinely makes a difference to patients’ experience [3].

In 2013, a global survey revealed that EBD projects had either been implemented, or were being planned in more than 60 health care organisations, in countries that include Australia, Canada, England, the Netherlands, New Zealand, Sweden, and the United States [3]. Projects focussed on a range of clinical services such as cancer, drug and alcohol treatment, emergency services, and mental health [1, 3]. For example, a pilot program in Australia investigated patients’ and caregivers’ experiences of emergency department care. Patients, their caregivers, clinicians, and administrators were invited to speak about their health care views and experiences, and to explore the implications of these views and experiences for how to redesign health care work [4].

There is a growing body of evidence showing the effectiveness of the EBD approach in terms of service improvement. For example, an evaluation of an EBD project across Guy’s and St Thomas’, and King’s College Hospital NHS Foundation Trust found that 22 months after the initial implementation of 56 co-design solutions, 66% had been sustained [5]. When considering the success of patient and public involvement within the EBD approach, a 2013 international survey found that 37 of the 41 respondents who had completed an EBD project said that the approach had ‘really engaged patients’, and 26 said ‘it allowed discussion of difficult topics in a supportive environment’ [6]. However, to our knowledge the barriers and facilitators to achieving successful public involvement in EBD projects have not been evaluated.

In 2013, Manchester Clinical Research Facility (MCRF) set up a Patient and Public Involvement and Engagement Group which has the aim of facilitating patient and public involvement and engagement across the MCRF. The MCRF is a generic adult and paediatric facility based at Central Manchester University Hospital’s NHS Foundation Trust which offers state of the art equipment, a team of specialist nurses and experience in supporting over six hundred commercial and academic studies across a wide range of disease areas. The MCRF Involvement and Engagement Groups has four public contributors, two students from a Greater Manchester high school and two adult public contributors who were recruited by advertisement. All were selected due to having a great interest in healthcare and in clinical research and both of the adult public contributors have experience as patients and carers. The work of this group reflects the fact that patient and public involvement is fundamental to the National Institute for Health Research (NIHR) strategy, and is a significant part of the funding requirements and strategy for NIHR CRFs and Biomedical Research Units. The Involvement and Engagement group consists of six staff from the Manchester Clinical Research Facility (MCRF), a PPI project manager with a background in qualitative research, two members of the public and two student representatives. When considering the patient experience at MCRF, an EBD approach was selected as the team wanted an approach that allowed public contributors and staff to work together in a collaborative fashion to improve the patient experience of the MCRF. Members of the Involvement and Engagement Group (including public contributors) attended quality improvement training which was focused on adopting an EBD approach to quality improvement. Following this training the Involvement and Engagement Group discussed potential approaches to exploring patient experience at the MCRF e.g., patient satisfaction surveys and they felt that adopting an EBD approach was the most appropriate way of approaching this work to ensure that all stakeholders (patients, public and staff) had ownership over the process. An EBD project was felt to provide an opportunity to improve our service by taking a bottom up approach to highlight what is really important to the patient rather than focusing on pre-determined questions.

This paper evaluates the EBD methodology with respect to how effectively public contributors at the MCRF were involved in the process, highlights good practice in public involvement in EBD and areas in need of improvement for other organisations who wish to replicate this approach [7]. This is important as the promotion of patient and public involvement activity in research and patient experience, rather than its evaluation, has been the focus of much of the published literature [8].

## Methods

This paper evaluates the processes that are used to involve public contributors to a clinical research facility in the co-design of an EBD project.

### Context

In May 2015 staff and public contributors from the Involvement and Engagement Steering Group attended a training session run by the Advancing Quality Alliance (AQuA) on Evidence Based Design. All four public contributors to the MCRF Involvement and Engagement Group agreed to be involved in this work. All had been members of the MCRF Involvement and Engagement Group for at least six months when this work was being undertaken. Therefore, they had considerable experience of discussing patient focused issues in relation to the MCRF. One of the working age public contributors also had experience of taking part in clinical trials.

After establishing a project lead (KB), the group decided to collect ‘stories’ from patients and carers from a range of trials to get an in-depth understanding of their experiences and identify areas for improvement. Public contributors were involved at all stages of this project including

- Making the decision regarding the choice of EBD as the method for this work
- Leading on the development of topic guides to be used in the collection of patient stories. This included: 1. Thinking through the patient journey when attending the MCRF, 2. Mapping the different stages of being involved in a research trial and 3. Thinking of the emotions that patients may feel at each point as prompts for collecting patient stories (http://ebdapproach.org/drupal/resources; Additional file 1: Appendix 1. This ‘experience questionnaire’ was designed to be used as a guide during the interviews to prompt interviewees to think about different points of their journey if necessary.
- Developing the recruitment process, considering what might be the best options for recruitment from a patient perspective
- Working age public contributors led the observations element of the project
- Student public contributors analysed the interview data and developed the first draft of the action plan
- Finally all public contributors helped to prioritise actions on the basis of the action plan findings

### Project process

Patients and carers were recruited by clinical research nurses at the MCRF who distributed information sheets to those visiting the MCRF and asked them if they would like to take part in this project. The MCRF has a large number of clinical trials running at any one time, and we therefore attempted to interview a wide range of patients taking part in a number of different trials (including healthy volunteers). If the patient or carer agreed to take part, KB asked them to sign the consent form and then conducted the interview on the same day. Participants lead the interview after they were asked about their experience of MCRF, focussing specifically on their emotions during this time. Prompts were only offered if necessary, allowing each participant to tell their own personal story; discussing any part of their experience that they felt was important to them. Five people from the MCRF (four patients and one healthy volunteer) and five people from the Children’s CRF (CCRF; four parents and one child) were interviewed between January and March 2016. All interviews were audio recorded and transcribed verbatim.

Alongside the interviews, two public contributors conducted an observation of the MCRF and CCRF to see how the facilities and interactions would appear to patients and the public. An experienced qualitative researcher (KB) provided a training session for the public contributors using materials from AQuA which outlined observation techniques (http://ebdapproach.org/drupal/resources; Additional file 1: Appendix 2). One public contributor also had previous experience of performing observations of NHS facilities.

The observations and interview transcripts were analysed by the student public contributors and KB. KB conducted face to face training with the young people in thematic analysis techniques before completing the analysis. Therefore public contributors received training to support them effectively in their role within the project. Therefore, not all received identical training as they were involved in slightly different aspects of the project. The emphasis of all of the training provided was on their being no ‘right or wrong’ answers and that all views and perspectives were valid.

The data for the MCRF and CCRF were analysed separately, highlighting positive statements and suggestions on how to improve the patient experience. Interview transcripts and observation notes were read, annotated, and categorised independently to increase reliability of the analysis [9, 10]. Main categories were then compared across interviews and reintegrated into common themes [11]. It was agreed that theoretical saturation across the data sets was achieved when no new themes emerged during the final interviews.

The final analysis was agreed through discussion and then presented to the Involvement and Engagement Steering Group at a bi-monthly meeting. All group members were sent the data, a list of themes and supporting quotes a month in advance of the meeting, and had the opportunity to challenge the analysis or highlight any additional themes. No changes were made to the analysis and all group members were in agreement on the final analysis. One explanation for this may have been that public contributors felt that they had been listened to throughout the process and that they had been able to shape the project from the start.

In line with the EBD approach, the results were used to develop an action plan with the aim of improving the patient experience at MCRF (template is available from authors on request). The group then worked through this action plan, highlighting priority areas and points that were not feasible to action. A traffic light system was then assigned to the remaining points, indicating ‘quick fixes’ (green), actions that were achievable within six months (orange) and long term projects (red). The action plan was structured around creating improvements in areas such as information provision and transport and directions to the facilities. Therefore, initial suggestions from the action plan have been implemented such as ensuring leaflet stands are re-stocked and information is updated on notice boards; and improving directions and signage to the CCRF. The wealth of positive feedback was shared with staff members via a staff newsletter.

## Box 1: Summary of the EBD approach used by MCRF

**Table:** 

1. Aqua training for staff and public contributors. 2. A project lead was established. 3. The group co-designed the project in terms of the objectives, the methodology and project timelines. 4. The group co-produced an experience questionnaire, information sheets and consent forms. 5. Patients and carers were recruited when visiting the MCRF and a member of staff collected their ‘patient stories’ on the same day. 6. Public contributors were trained by an experienced qualitative researcher in observation and qualitative data analysis techniques. 7. Two public contributors conducted an observation of the MCRF and CCRF. 8. The observations and interview transcripts were analysed by two young peoples’ public contributors and KB. 9. The Involvement and Engagement Steering Group were given the opportunity to challenge the analysis or highlight any additional themes. 10. The results were used by the Involvement and Engagement Steering Group to develop an action plan with the aim of improving the patient experience at MCRF. 11. Positive feedback was shared with staff members via a staff newsletter. 12. A member of staff developed and analysed a questionnaire to evaluate the public involvement process within the project. The group aimed to disseminate the findings of this evaluation in an academic journal. The two student public contributors contributed to the writing of this paper.	

### Evaluation

Once the first draft of the action plan had been completed, KB developed an open ended evaluation questionnaire (Additional file 1: Appendix 3) from a review of the literature and discussions within the Involvement and Engagement Steering Group. The open ended nature of the questions allowed participants to express their opinions freely rather than responding to tick boxes or satisfaction scales. The questionnaire was circulated to staff and public contributors from the Involvement and Engagement Steering group who were involved in the project. Questions included whether each individual felt that they were able to shape the project from the beginning; if they received adequate training; whether they had enough time to complete each task; and how well they thought the group worked together to develop the action plan. The questionnaire also asked participants to reflect on what worked well; the benefits of being involved in the project and how they thought the project could have been improved in terms of the involvement of public contributors.

KB completed a thematic analysis of the data by comparing the main categories across the completed questionnaires and integrating them into common themes [11]. The aim of this evaluation was to provide recommendations for best practice in effectively involving public contributors in an EBD approach.

## Results

Feedback on the evaluation questionnaire was received from seven out of the ten individuals involved in this project (four public contributors and three staff members). Five themes were identified from the data: building relationships and shaping the project; the impact of training; the time provided for each task; developing the action plan; and the personal benefits of EBD.

### Building relationships and shaping the project

Public contributors reported that they were able to influence the design of the project from the beginning. All comments and suggestions were respected and actively discussed within the group. Both the adult and student contributors felt that they were able to challenge the views of the staff in the group and that their ideas were valued and listened to throughout the project. Differing opinions were debated and the group was able to compromise on the best way forward. The respect for everyone’s opinions meant that staff and public contributors built successful working relationships:

I always felt as though any contribution I offered would be likely to be both welcomed and acted upon… the meetings are always open and encouraging of independent views. (Public contributor 1)

Patients and staff in the group worked very well together, a good group with various opinions. (Staff 1)

Staff from varying backgrounds, including nursing, management and research were also able to contribute to the project and felt that their individual skills were valued and utilised:

I felt that we had the opportunity to design this project from scratch. One staff member had expertise in qualitative research which helped with training for interviews and the analysis of the data, while others brought knowledge of what had already been done in MCRF around patient experience. Public contributors brought new ideas and knowledge on how to complete successful observations. We worked together in an open way where everyone’s opinions were valued and we had time to debate the best way forward. (Staff 3)

### The impact of training

Public contributors received training to provide them with the skills to fully engage with this project. This included the opportunity to attend the AQUA Experience Based Design (EBD) Training day with staff members. This training provided an overview of the EBD methodology, ensuring that everyone had the same level of understanding before the project started:

Staff and patients were able to attend AQUA training days and we also had a visit from Helen Baxter who ran the sessions to help us design the project. I thought that this gave us the information and skills to do the project. Helen was also on hand if we needed any further support (Staff 3).

Public contributors were also given the opportunity to volunteer to receive training to take part in an observation task of the MCRF and CCRF or complete the analysis of the patient interviews. The training was necessary to provide the skills and confidence to complete these tasks. In terms of the observation task, the public contributors felt satisfied with the level of information they received. The AQUA guidance was used to provide some pointers. This was not too structured in order to allow the public contributor to focus on the task rather than follow complicated instructions:

I was given instruction on where the Children’s CRF was located and some sample questions/issues to give me some idea of the potential scope of my brief. Although initially daunted, it did prove to be useful preparation for me to rely on what I was able to observe for myself rather than what I might interpret people expected me to focus on. This was a very reasonable and valid way to provide instruction. (Public contributor 1)

I felt very confident when performing the observation. (Public contributor 2)

It was noted that each person wrote up their notes in a different way. One member had extensive experience in observing NHS services and therefore provided significantly more detail. Both accounts were very informative and fed into the action plan; however, a reporting template may have improved consistency of reporting:

The two observations were very different so perhaps by giving more information to the observers so they were done to the same level of detail. (Young person public contributor 2)

In terms of training for the thematic analysis, the young public contributors who volunteered for this role received verbal and written instructions including examples of analysis techniques. Overall, this was an adequate amount of input for these individuals:

I feel that the training I received on this front was sufficient for the task. [A member of staff] showed us how to go about analysing… the guidance given to me made me feel confident, combined with the fact that there were several people doing the same job meaning if I missed something someone else would pick up upon it… it [was made] clear had I needed any other guidance I could have asked for it. (Young person public contributor 1)

However, one young person did suggest that one to one support when completing the analysis may have improved the experience:

At first I felt slightly unsure but I quickly became confident as I got into the analysis. Perhaps going through a paragraph together as an example during the training could have made me more confident, however it didn’t take me long become confident with the process by myself. (Young person public contributor 2)

In addition, there was some inconsistency in the level of feedback expected by public contributors on their work. It is important to regularly check that the public contributor has received all the support they need. This includes after the task has been completed:

I cannot recall receiving any feedback on my analysis. It was used alongside others so this made feedback slightly less practical however it would have been nice to have some reassurance I did things along the right lines. (Young person public contributor 1)

Yes the feedback was very supportive and I don’t think more detailed feedback was needed. (Young person public contributor 2)

Training increased the confidence of the patient and public representatives, enabling them to fully engage with the project. This in turn helped to create a balance of power between all members of the group:

I think the training really helped to ensure that every member of the group felt that they had the skills to contribute. Everyone understood the role that the patient and public members of the group had in the project and the importance of their input. This helped to break down any power imbalance that you can sometimes get within these groups. (Staff 3)

### The time provided for each task

Public contributors and staff felt that adequate time was devoted to this project. This increased satisfaction as they did not feel overwhelmed or overstretched during the project:

When training public contributors I felt that there was enough time for them to get to grips with the task and do a thorough job of the task itself. (Staff 3)

The young public contributors, who attended the Involvement and Engagement Steering group, also valued the ability to see the project through from start to finish:

the benefits were I found out more about the way projects are designed in the NHS and the EBD approach, having not been involved in such a venture before. It was also very rewarding to see the project come to fruition before the end of my tenure on the PPI/E committee. (Young person public contributor 1)

However, by providing adequate time for each task, some members of the team felt that the project took longer than expected. For example, those who were not involved in the analysis of the data had to wait for the public contributors to be trained and complete the task before we could move onto developing the action plan. This could be frustrating for some members and more regular updates on how certain aspects of the project were progressing may have maintained a feeling of momentum:

I’ve had to learn to be more patient – things seem to happen very slowly! (Public contributor 1)

Furthermore, personal commitments restricted involvement in activities public contributors would have liked to gain experience in:

I would have liked to have been a part of the interviewing stage myself but due to the limitations of my school timetable this was not possible although it was always made to clear to me that I could have received training had I wanted it and could have been an active member of this process had I been able. (Young person public contributor 1)

### Developing the action plan

The action plan was informed by the observations of the MCRF and CCRF, and the patient stories. The individuals who completed the observations felt that all their work and suggestions had informed the development of this plan. The young public contributors who completed a thematic analysis of the patient data also fed into the writing of the action plan. These individuals felt that their contributions were valued and that they were able to shape the final document:

My observations were valued, yes, and acted upon. (Public contributor 2)

Yes my themes were included in the action plan as were comments that I had included in my analysis so I felt that my work shaped the results. (Young person public contributor 2)

The draft action plan was then presented to the rest of the group after they had had three weeks to look at the interview transcripts and observation data. The members of the group all reported that this was a good way to present the action plan and that they had enough time to provide feedback:

…it was presented in a table and we talked through each step allowing time for any questions to be asked so we could each fully understand it. I could not suggest any way in which to improve this. (Young person public contributor 1)

When presenting the action plan to the group it was made clear that this was a draft and that the discussions within the meeting would inform the final document. Although the development of the plan was led by one staff member; everyone felt ownership of the final document:

Yes, any comments were always welcomed and suggestions were included and the action plan was amended based on the group’s opinions. (Young person public contributor 2)

The interview data informed the draft action plan and patient reps and staff worked together to analyse that data. All findings were put into the action plan and then we worked together as a group to discuss what actions were feasible and the timeframe for these actions. (Staff 3)

### The personal benefits of EBD

All public contributors are members of the MCRF Involvement and Engagement Steering Group and had attended bi monthly meetings for six months previous to the start of the project. They had therefore worked together to advise on the day to day work of MCRF. However, this was the first project that public contributors felt that they could get involved with from the very beginning and therefore felt a sense of ownership over the work. They valued being able to take part in a project with a tangible outcome and to be directly involved in collecting and analysing data:

So far this is the only practical matter in which I’ve had any involvement so it has been personally rewarding to have finally been able to make a practical ‘hands-on’ contribution. (Public contributor 1)

The members of the team reported that they found that the EBD project was a interesting and worthwhile initiative. Working to improve patient experience was therefore very rewarding for both the staff and public contributors:

It felt like such an achievement to help to improve the patient experience. The interviews with patients gave a real insight on what was important to them, and also what we were doing right which gave the staff a boost. (Staff 3)

This project also provided opportunities for staff and public contributors to learn new skills. For example, the young person contributors presented a poster about the project at the annual UKCRF conference, and developed skills in analysis and project design. These new experiences increased their confidence and were also beneficial for their future careers:

I had never been involved in anything similar to this before so it improved my analytical skills, my ability to pick out themes and my ability to digest large volumes of information… Being involved in the analysis increased my understanding of how patients experience illness and healthcare which will be very useful in my future career. To read such personal accounts from patients is a privilege and provides a unique viewpoint to learn from… Being able to present the poster for this project at the UKCRF conference and being involved in the paper will also be very helpful in my future career… I have also developed my team working skills by being involved in this project. (Young person public contributor 2)

It has been beneficial professionally, working in a group improving the service for the future… I have built confidence in a group setting and have broadened my knowledge of PPI. (Staff 1)

Overall, this was a positive experience for both staff and public contributors, all members of the team stated that they would take part in a similar project in the future:

Yes, taking part in the project has been very interesting and helped my future career so I would definitely be interested in being involved in something similar. (Young person public contributor 2)

I would like to take part in a similar project, it’s been interesting to be involved in new ideas and change. (Staff 1)

## Discussion

This paper provides an evidence base on effective ways to involve staff and public contributors in an Experience Based Design (EBD) project. Previous work has evaluated the impact of the EBD approach on NHS services [6, 12, 13], but has not explored how to effectively involve public contributors within these projects, or highlight the personal benefits for those involved [14].

Staff and public contributors were asked to evaluate their experience of co-designing an EBD project. This involved completing training on the EBD process; working together to co-design the project; completing a thematic analysis of qualitative interview and observation data; and using this data to inform an action plan. We have used the insights of staff and public contributors to generate recommendations which may help others engage in successful EBD projects (Table 1). This should be considered alongside existing guidance on PPI [15]. We have also developed a brief checklist to inform planning patient and public involvement in EBD projects (Table 2).

**Table 1:** Lessons learnt: working with public contributors on an Experience Based Design project

1. Spend time building relationships within the group in order for members to feel comfortable expressing their opinions. This could include an ice breaker session or choosing team members who have worked together on previous projects.
2. Identify the different skills that each member can bring to the group and utilise these. This can help individuals to feel valued and useful.
3. Negotiate the level of involvement that is suitable for the public contributors and the specific project.
4. Training for staff and public contributors on EBD provides everyone with the information they need to co-design the project. This knowledge helps to address any power imbalance that may exist between staff and public contributors.
5. Identify additional training needs within the group. This may include written and verbal training on analysis or observation techniques. This allows staff and public contributors to be involved in all aspects of the project.
6. Consider the previous experience and level of education of the individual receiving the training and adapt the content accordingly.
7. Provide adequate time for each stage of the EBD project to ensure that public contributors and staff do not feel overwhelmed or overstretched by the workload. Discuss personal commitments and other deadlines that may impact on the EBD project when developing a timeline at the beginning of the project.
8. Develop a template for the observation task to increase the consistency of the data gathered.
9. Provide regular updates on how certain aspects of the project are progressing in order to maintain a feeling of momentum for all members of the team.
10. Identify an appropriate amount of time for members of the team to explore the patient stories and observations before holding a session to discuss the action plan.
11. A face to face meeting is required to discuss the findings of the project and work together to suggest actions to improve the service. We found that a group of four public contributors and six staff was a good number to allow a meaningful discussion.
12. Feedback should be given to those who complete any analysis or observation tasks, highlighting good quality work and any further training needs. Clear feedback is also required on how the public contributors have fed into and informed the action plan.
13. Thank public contributors for their involvement and highlight the value of their input.
14. Continue to involve and update public contributors on the impact of the action plan and the dissemination of the findings.

**Table 2:** Checklist for planning patient and public involvement in EBD projects

1. Provide public contributors with ample opportunities to influence the design and implementation of the project from beginning to end
2. Provide public contributors with appropriate training and support tailored both to their and the projects needs and regularly check with public contributors that the support provided continues to be fit for purpose
3. Manage the project team’s expectations regarding the amount of time that certain aspects of the project are likely to require
4. Provide ample time for data analysis to enable action plans resulting from the EBD work to be fully informed by all project partners interpretations of data
5. Emphasize to all project partners (both staff and public contributors) the likely benefits of the project both personally and professionally to them

### Comparisons with existing literature

Acting as a public contributor to healthcare research projects can often require patients and the public to draw on their personal experience to suggest ways that could improve a project or service. However, some public contributor roles, such as involvement in an EBD project, can also require skills in project design, interviewing, observation and data analysis. This therefore goes beyond personal experience and asks the patient to invest time in training to support their contribution [8, 15–18]. The staff and public contributors in the current EBD project highlighted the personal benefits that they gained from this experience. This ranged from a feeling of ownership and pride as they were able to shape the project from the start, to personal skills such as increased confidence in groups; a greater understanding of public involvement; and analysis skills. Every member of the group stated that they would take part in a similar project again and felt that their contribution was meaningful and beneficial for the patients who use MCRF.

Buck et al. [7] report that it can be difficult to find and engage patient and public representatives with an interest in and understanding of service improvement. However, the Involvement and Engagement Steering Group which led this EBD project had already recruited articulate and educated members of the public, who were fully engaged in the topic as they had previously worked on projects within MCRF. The young people on this group were also recruited from a local grammar school, and were aiming to study medicine at University. Enany et al. [19] state that public contributors with higher levels of education may be prone to over identify with the perspectives of staff rather than challenging them. This may partly explain why there was a low level of disagreement within the group. Other factors such as EBD training, providing sufficient time for each task, the previous experience of public contributors, and the fact that the group had worked together before may also explain the low level of conflict in the group. In the future, we believe that it would be beneficial to develop approaches to identifying, involving and supporting public contributors from a wider range of socio-demographic backgrounds to be involved in EBD projects, which may lead to a greater diversity of opinion being expressed. This may involve providing support to all team members (staff and public contributors) in articulating your ideas in a group setting, listening to and responding to conflicting opinions and how to work effectively in a group.

Staley [8] refers to the challenge of ensuring that involvement is meaningful and not tokenistic. This issue points to a ‘know-do’ gap, whereby the importance and value of patient and public involvement in the ‘ideal’ world contrasts with what is achievable when implementing patient and public involvement in practice [20]. In the current project, not all public contributors and staff were able to commit to getting involved in gathering patient stories, completing observations, analysing the data and developing the first draft of the action plan. Some members of the group would have liked to be more involved but had other pressures on their time. These individuals therefore relied more on their personal or work experience to make comments and suggestions on the action plan rather than analysing the data [21]. By considering what is achievable in a given timeframe the group can work together to allocate tasks and attempt to work flexibly so patients and members of the public can be involved as possible [7, 22].

### Strengths and limitations

To our knowledge, this is the first study to evaluate EBD methodology in terms of achieving successful public involvement. The feedback from the staff and public contributors can be used to help others plan how to effectively involve and work with public contributors within EBD projects in their organisation. This follows the recommendations of the Public Involvement Impact Assessment Framework (PiiAF), which emphasises the value of well thought-through planning before implementing public involvement as well as the evaluation of its impact [23].

The public contributors on this project were highly committed to seeing the project through from the beginning to the end. It is important to consider the impact of payment on this level of commitment. In line with INVOLVE guidelines, the Public contributors were paid £75 for attending bi-monthly Involvement and Engagement Steering Group meetings to design and deliver this project [15]. However, the observations, analysis and contributions to the writing of this paper were completed on a voluntary basis. The student public contributors were also not paid for their positions. The personal benefits reported by the public contributors in this evaluation also contributed to retention.

When reflecting on the level of control in EBD process, it is important to recognise that a member of staff managed the project and trained the public contributors in observation and analysis techniques. The information included in these training sessions was very much in the control of the staff member. However, the public contributors were then able to complete the analysis and observations, which meant that the level of control when developing the action plan was shared equally between staff and public contributors. Although we did not experience a power imbalance within the group, it is important to be aware of the distribution of power when co-designing a project. Robinson et al. [24] found that it can be difficult for researchers to relinquish control and work with patients when analysing data. This suggests that EBD training should also include further guidance on how to meaningfully involve patients in the analysis of patient stories and observation techniques, and what to do when they have contrasting views about data. Noe et al. [25] also highlights the importance of developing training that engages with the diversity of learners’ needs and is meaningful from their perspective.

It is important to note that this evaluation was conducted by a staff member who was involved in the EBD project. Public contributors may have been more critical if they were asked to evaluate the study by a third party. However, it is important to note that public contributors did provide some critical comments so it seems unlikely that they were unable to articulate their own perspectives [21]. It is also important to highlight that three out of the six members of staff who participated in this project did not complete the evaluation questionnaire.

## Conclusion

The EBD approach was successful in developing strong and productive working relationships between staff and public contributors when designing and implementing a project that aimed to improve the patient experience at MCRF. Public contributors and staff reported that the training provided, the time to develop a project from scratch and a mutual respect for differing opinions and healthy debate made the project both professionally and personally rewarding. This evaluation highlights the lessons we have learnt on how to achieve successful involvement of public contributors within EBD, and can be used to encourage a more consistent approach to EBD in other organisations. However, it is important to note that there is a need for flexibility in order to accommodate the unexpected and respond to opportunities and difficulties as they arise.

It is worth noting that shortly after this work was undertaken that the MCRF needed to submit for further NIHR funding to continue its work. This has meant that further implementation of the action plan needed to be paused until the funding decision was known. The MCRF has recently been successful in achieving this funding. Therefore, the nature of the future involvement of public contributors in implementing the action plan will shortly be discussed and decided upon. The initial ideas are for the Involvement and Engagement Steering Group at MCRF to repeat the public contributor lead observations of MCRF on a bi-annual basis to monitor whether the actions detailed in the action plan have been completed and maintained. These observations will also add to the action plan so that this is an evolving and active document. However, it is possible that further changes may be made to the approach following the recent successful funding decision. Finally, the group also aim to share the benefits of this approach with other CRFs and researchers and offer guidance where required.

## Additional file

Additional file 1: Appendix 1. AQUA template for an experience questionnaire. Appendix 2. AQUA observation guide. Appendix 3. Evaluation questionnaire. (DOC 80 kb)

## References

[1] Baxter H, Mugglestone M, Maher L. The EBD Approach. Evidence Based Design. Using patient and staff experience to design better healthcare services. Concepts and case studies. Institute for Innovation and Improvement. 2009. https://www.hqsc.govt.nz/assets/Consumer-Engagement/Partners-in-Care-Resource-page/Experience-Based-design-Concepts-and-Case-Studies-January-2010.pdf. Accessed 21 Apr 2017.

[2] Bate SP, Robert G. Bringing user experience to health care improvement: the concepts, methods and practices of experience-based design. Oxford: Radcliffe Publishing; 2007 (https://www.crcpress.com/Bringing-User-Experience-to-Healthcare-Improvement-The-Concepts-Methods/Bate-Robert/9781846191763). Accessed 21 Apr 2017.

[3] The Kings fund. 2013. https://www.kingsfund.org.uk/projects/ebcd. Accessed 21 Apr 2017.

[4] Iedema R, Merrick E, Piper D, Britton K, Gray J, Verma R, Manning N (2010). Codesigning as a discursive practice in emergency health services: the architecture of deliberation. J Appl Behav Sci.

[5] King’s College London. An evaluation of the spread and sustainability of experience-based co-design. 2012. [online]. Available at: http://www.kcl.ac.uk/nursing/research/nnru/research-programme/Organisations,environmentandwaysofworking/An-evaluation-of-the-spread-and-sustainability-of-experience-based-co-design.aspx. Accessed 21 Apr 2017.

[6] The King’s Fund. Is experience-based co-design for you?. The growing evidence base. 2016. [online]. Available at: https://www.kingsfund.org.uk/projects/ebcd/experience-based-co-design-introduction. Accessed 21 Apr 2017.

[7] Buck D, Gamble C, Dudley L, Preston J, Hanley B, Williamson PR, Young B, EPIC Patient Advisory Group (2014). From plans to actions in patient and public involvement: Qualitative study of documented plans and the accounts of researchers and patients sampled from a cohort of clinical trials. BMJ Open.

[8] Staley K (2009). Exploring impact: Public involvement in NHS, public health and social care research.

[9] Peters S (2010). Qualitative research methods in mental health. Evid Based Ment Health.

[10] Bayliss K, Riste L, Band R, Peters S, Wearden A, Lovell K, Fisher L, Chew-Graham C (2016). Implementing resources to support the diagnosis and management of Chronic Fatigue Syndrome/Myalgic Encephalomyelitis (CFS/ME) in primary care. A qualitative study. BMC Fam Pract.

[11] Huberman AM, Miles MB, Denzin NK, Lincoln YS (1994). Data management and analysis methods. Handbook of Qualitative Research.

[12] Tsianakas V, Robert G, Maben J, Richardson A, Dale C, Wiseman T (2012). Implementing patient centred cancer care: using experience-based co-design to improve patient experience in breast and lung cancer services’. J Support Care Cancer.

[13] Piper D, Iedema R (2010). Emergency Department Co-Design Program 1 Stage 2 Evaluation Report.

[14] Robert G, Ziebland S, Calabrase J, Coulter A, Locock L (2013). Participatory action research: using experience-based co-design (EBCD) to improve health care services’. Understanding and using experiences of health and illness.

[15] INVOLVE (2012). Developing training and support for public involvement in research.

[16] Snape D, Kirkham J, Preston J, Popay J, Birtten N, Collins M (2014). Exploring areas of consensus and conflict around values underpinning public involvement in health and social care research: a modified Delphi study. BMJ Open.

[17] Stewart R, Liabo K (2012). Involvement in research without compromising research quality. J Health Serv Res Policy.

[18] Boote J, Baird W, Beecroft C (2010). Public involvement at the design stage of primary health research: a narrative review of case examples. Health Policy.

[19] Enany NE, Currie G, Lockett A (2013). A paradox in healthcare service development: professionalization of service users. Soc Sci Med.

[20] Ward PR, Thompson J, Barber R (2010). Critical perspectives on ‘consumer involvement’ in health research: epistemological dissonance and the know-do gap. J Sociol.

[21] Bayliss K, Starling B, Raza K, Johansson EC, Zabalan C, Moore S, Skingle D, Jasinski T, Thomas S, Stack R (2016). Patient involvement in a qualitative meta-synthesis: Lessons learnt. Res Involvement Engagement.

[22] Dudley L, Gamble C, Allam A, Bell P, Buck D, Goodare H, Hanley B, Preston J, Walker A, Williamson P, Young B (2015). A little more conversation please? Qualitative study of researchers’ and patients’ accounts of training for patient and public involvement in clinical trials. Trials.

[23] PiiAF Study Group. The Public Involvement Impact Assessment Framework: executive summary. http://piiaf.org.uk/documents/exec-summary-0114.pdf. Accessed 21 Apr 2017.

[24] Robinson L, Newton J, Dawson P (2012). Professionals and the public: power or partnership in health research?. J Eval Clin Pract.

[25] Noe RA, Tews MJ, McConnell Dachner A (2010). Learner engagement: A new perspective for enhancing our understanding of learner motivation and workplace learning. Acad Manag Ann.

