# Supplementary material for: Working with public contributors to improve the patient experience at the Manchester Clinical Research Facility: an evaluation of the Experience Based Design approach
Source: Res Involv Engagem. 2017 Apr 26;3:10. doi: 10.1186/s40900-017-0059-x (PMC5611576; doi:10.1186/s40900-017-0059-x)
Supplement: Additional file 1: — Appendix 1. AQUA template for an experience questionnaire. Appendix 2. AQUA observation guide. Appendix 3. Evaluation questionnaire. (DOC 80 kb) [file 40900_2017_59_MOESM1_ESM.doc]

**Additional file 1**

**Appendix 1: AQUA template for an experience questionnaire**

**This experience questionnaire will help you think about how you feel at different stages in your journey.**

**How did you feel?**

Circle the words that best describe your feelings at each stage, or write your own words below

**Why?**

We’d like to know why you felt like this. Was it friendly staff, a nice conversation, or a long wait?

happy

supported

safe

good

comfortable

in pain

worried

lonely

sad

**_______________**

**Write your own**

**words here**

happy

supported

safe

good

comfortable

in pain

worried

lonely

sad

**_______________**

**Write your own**

**words here**

happy

supported

safe

good

comfortable

in pain

worried

lonely

sad

**_______________**

**Write your own**

**words here**

happy

supported

safe

good

comfortable

in pain

worried

lonely

sad

**_______________**

**Write your own**

**words here**

happy

supported

safe

good

comfortable

in pain

worried

lonely

sad

**_______________**

**Write your own**

**words here**

happy

supported

safe

good

comfortable

in pain

worried

lonely

sad

**_______________**

**Write your own**

**words here**

happy

supported

safe

good

comfortable

in pain

worried

lonely

sad

**_______________**

**Write your own**

**words here**

**Arriving/**

**Checking In**

**Information**

**Waiting**

**Going to Theatre**

**Recovery**

**Check Ups**

**Leaving**

**Write your own**

**words here**

**Write your own**

**words here**

**Write your own**

**words here**

**Write your own**

**words here**

**Write your own**

**words here**

**Write your own**

**words here**

**Write your own**

**words here**

**Appendix 2: AQUA observation guide**

**Observation Guide**

Observation is a key part of discovering and understanding patient and families and carers,

and staff experience.

The first thing you can do is **take a step back** and look at what is happening through

**fresh eyes**.

Next time you walk into your department or clinic, just have a look round and see the

environment for the ‘first time’.

Check the signage, look at the notice boards, what behaviour/feelings do the seating

arrangements encourage? Does it feel welcoming? Are the receptionists ready to greet you

or are they ‘busy’ with other activities.

**Sit and watch** what actually happens.

What do you **hear** and **smell?**

**Don’t jump to conclusions (or solutions).** Observation is a part of the understand

phase along with the narratives from service users.

**Ask people to show you what they do** rather than tell you…it is important to see what

happens, not what people think happens.

**Keep an open mind**. It is important to understand someone’s experience is their truth –

so don’t try and correct it, use it as a part of your data to understand the service better.

Look out for **pauses, obstacles and body language.** What do people care about by the

way they behave and how have they adapted the environment to make it work belter for

them?

**Look out for surprises!**

For more information visit:

www.ebdapproach.org

**Appendix 3: Evaluation questionnaire**

**Evaluation: Experience Based Design Project**

**Questions for patients and staff involved in the EBD project (Everyone)**

**Did you feel like you were able to shape this project from the beginning** (I.e. were your views taken into consideration at each stage?)

**Did you receive adequate training for this project?** (Please give details about what you liked about this training and what could be improved)

**Is there any further training that you would have liked to receive?** (How would you like this to have been delivered?)

**Were you able to contribute to the project in the way you wanted to?** (E.g. commenting on the patient information sheets and consent forms, observations, analysis, developing the action plan etc?)

**Did you have enough time to complete any tasks that you were involved in?** (If not, how much time do you think would be appropriate?)

**Was the draft action plan clearly presented to the group?** (If not, would you have preferred to receive this information in a different way?)

**Did you feel like you had the opportunity to contribute to/share your views about the content of the action plan and how it was delivered?**

**Do you think the patients and staff in the group worked well together?** (If not, what could be improved?)

**What were the benefits of being involved in this project?** (Personal or professional)

**Have you developed any new skills?**

**How do you think this project could have been improved?**

**Would you like to take part in a similar project again?**

**Any further comments?**

**Questions for patient reps involved in the observation.**

**How did you feel about the training for this activity?** (What did you like/what could be improved?)

**Did you feel confident when performing your observation?** (What could have helped you to feel more confident?)

**How much time did it take to complete this activity?** (Did you feel like this was enough time to complete the observation properly?)

**Did you receive adequate feedback from the rest of the team on your observations?**

**If not, what feedback would you have liked?**

**Did you think your observations were valued?** (I.e. were they included in the action plan or was an explanation provided if it was not?)

**What were the benefits of being involved in this activity?** (Personal or professional)

**Have you developed any new skills?**

**How do you think this process could have been improved?**

**Any further comments?**

**Questions for patient reps involved in the analysis of the data.**

**How did you feel about the training for this activity?** (What did you like/what could be improved?)

**Was the written information provided as part of your training clear?** (How could this be improved?)

**Did you feel confident when performing your analysis?** (What could have helped you to feel more confident?)

**How much time did it take to complete this activity?**

**Did you receive adequate feedback on your analysis?**

**If not, what feedback would you have liked?**

**Did you think your analysis shaped the results of the project** (I.e. were your themes included in the action plan or was an explanation provided if they were not?)

**What were the benefits of being involved in this activity?**

**Have you developed any new skills?**

**How do you think this process could have been improved?**

**Any further comments?**
